# Effectiveness of Platelet Function Analysis-Guided Aspirin and/or Clopidogrel Therapy in Preventing Secondary Stroke: A Systematic Review and Meta-Analysis

**DOI:** 10.3390/jcm9123907

**Published:** 2020-12-01

**Authors:** Ann-Rong Yan, Mark Naunton, Gregory M. Peterson, Israel Fernandez-Cadenas, Reza Mortazavi

**Affiliations:** 1 School of Health Sciences, Faculty of Health, University of Canberra, Canberra 2617, Australia; Ann-Rong.Yan@canberra.edu.au (A.-R.Y.); Mark.Naunton@canberra.edu.au (M.N.); g.peterson@utas.edu.au (G.M.P.); 2 School of Pharmacy and Pharmacology, University of Tasmania, Hobart 7000, Australia; 3 Stroke Pharmacogenomics and Genetics Group, Neurovascular Research Laboratory, Hospital de Sant Pau, 08041 Barcelona, Spain; israel.fernandez@vhir.org; 4 Prehab Activity Cancer Exercise Survivorship Research Group, Faculty of Health, University of Canberra, Canberra 2617, Australia

**Keywords:** antiplatelet, aspirin, clopidogrel, ischemic stroke, TIA, platelet function analysis, antiplatelet therapy modification, secondary stroke prevention, high on-treatment platelet reactivity

## Abstract

Background: Antiplatelet medications such as aspirin and clopidogrel are used following thrombotic stroke or transient ischemic attack (TIA) to prevent a recurrent stroke. However, the antiplatelet treatments fail frequently, and patients experience recurrent stroke. One approach to lower the rates of recurrence may be the individualized antiplatelet therapies (antiplatelet therapy modification (ATM)) based on the results of platelet function analysis (PFA). This review was undertaken to gather and analyze the evidence about the effectiveness of such approaches. Methods: We searched Medline, CINAHL, Embase, Web of Science, and Cochrane databases up to 7 January 2020. Results: Two observational studies involving 1136 patients were included. The overall effects of PFA-based ATM on recurrent strokes (odds ratio (OR) 1.05; 95% confidence interval (CI) 0.69 to 1.58), any bleeding risk (OR 1.39; 95% CI 0.92 to 2.10) or death hazard from any cause (OR 1.19; 95% CI 0.62 to 2.29) were not significantly different from the standard antiplatelet therapy without ATM. Conclusions: The two studies showed opposite effects of PFA-guided ATM on the recurrent strokes in aspirin non-responders, leading to an insignificant difference in the subgroup meta-analysis (OR 1.59; 95% CI 0.07 to 33.77), while the rates of any bleeding events (OR 1.04; 95% CI 0.49 to 2.17) or death from any cause (OR 1.17; 95% CI 0.41 to 3.35) were not significantly different between aspirin non-responders with ATM and those without ATM. There is a need for large, randomized controlled trials which account for potential confounders such as ischemic stroke subtypes, technical variations in the testing protocols, patient adherence to therapy and pharmacogenetic differences.

## 1. Introduction

Recurrent stroke is a major concern in patients with an initial stroke or transient ischemic attack (TIA) [1,2,3]. On average, the cumulative rate of recurrent ischemic stroke/TIA is 5.4% at one year, 11.3% at five years [4] and as high as 43% within 10 years from an initial event [2,5]. In terms of increased chances of the short-term occurrence of stroke after a TIA event, there are differences in the literature. For example, a Norwegian prospective cohort study reported a 0.9% risk of having stroke within 7 days of a TIA [6]. On the other hand, in a population-based study in the UK, the reported risk for this time point was much higher (8.6%) [7]. Regardless of the magnitude of the reported risks, the risk is real, and recurrent events are a continuing challenge for patients and healthcare systems alike worldwide [2,8]. Therefore, there is an urgent need for effective strategies to prevent stroke recurrence both in the short- and long term.

Platelets have a key role in the development of atherothrombosis and thrombotic events such as ischemic stroke [9,10,11]. Antiplatelet medications reduce the absolute risk of thrombotic vascular events by 2% per annum, although they concomitantly increase the risk of major extracranial hemorrhage by 0.1% to 0.3% per annum [2]. Current clinical guidelines, for example the living Clinical Guidelines For Stroke Management, published by the Stroke Foundation (Australia), strongly recommend long-term antiplatelet treatment for all patients with ischemic stroke or TIA who are not receiving prophylactic anticoagulants [5]. However, antiplatelet treatments may be ineffective due to various reasons, such as poor patient adherence [12] or individual variations in the genes related to the pharmacokinetics or pharmacodynamics of antiplatelet drugs, which render these drugs non-effective or less effective in the body [13].

Aspirin (acetylsalicylic acid) irreversibly inhibits the bone marrow and blood megakaryocytes and platelets by acetylating the 529th amino acid of the enzyme cyclooxygenase 1 (COX-1), thereby blocking COX-1 from producing prostaglandin G_2_/H_2_, which is an essential substrate for thromboxane A2 (TXA_2_) synthesis [12]. Aspirin ineffectiveness (or resistance) can be attributed to a number of reasons including but not limited to the patient non-adherence, a blocked binding site on COX-1 due to interference by other drugs such as nonsteroidal anti-inflammatory drugs (NSAIDs), common variations (polymorphisms) of the COX-1 gene, non-platelet pathways for TXA_2_ production (e.g., biosynthesis by the monocyte/macrophage COX-2), non-thromboxane-dependent platelet activation (e.g., adenosine diphosphate (ADP)—dependent platelet activation), or an over-production of platelets by the bone marrow in response to stress (e.g., inflammation or infection) [14,15].

Clopidogrel is a pro-drug (inactive), which, following oral administration and absorption into the bloodstream, is activated in a two-step metabolic process by hepatic cytochrome P450 enzymes. The active thiol metabolite inhibits ADP-induced platelet activation by binding to the P2Y12 receptors on the platelet surface, thereby preventing the binding of ADP molecules (as platelet activators) to their normal receptors [15,16,17]. Common causes of clopidogrel resistance include patient non-adherence, inadequate dose or problems with intestinal absorption, inhibition of the cytochrome P (CYP) isoenzymes due to drug interactions (for example, inhibition of CYP2C19 by some proton pump inhibitors), increased platelet production and polymorphisms of CYP450 genes [15,17].

Antiplatelet resistance is commonly referred to as high on-treatment platelet reactivity (HTPR) or platelet non-responsiveness [18]. The overall prevalence of HTPR in ischemic stroke or TIA patients is reported to be 20–28% and 22–32% for aspirin and clopidogrel users respectively, with an estimated range of 5–10% resistance to both drugs in patients taking them simultaneously [19]. Numerous studies have reported associations between HTPR and adverse clinical outcomes. For example, Sabra et al. reported higher rates of HTPR in patients with acute ischemic stroke (AIS) than in healthy volunteers [20], while others highlighted similar differences in patients with recurrent stroke compared with those without a stroke recurrence [21,22]. Other studies have revealed an association between aspirin-HTPR in the initial stages of AIS with stroke severity and infarct volume [23,24,25], as well as the inflammation status [26]. HTPR could predict 72 h and 10-day early neurological deterioration [27,28], and 1-week early recurrent stroke lesions following the initial ischemic event [29]. These findings are suggestive of a higher risk of stroke recurrence in patients with HTPR. This view is supported by other studies [27,28,30,31,32].

Given the importance of effective antiplatelet treatments in the prevention of recurrent thrombotic events, there has been a long-lasting interest in the development of laboratory tests for assessing platelet function during antiplatelet treatment. Platelet function analysis (PFA) was initially introduced in the early 1960s by the late Professor G. V. R. Born of King’s College, London, based on the aggregation-related changes in the quantity of light transmission from platelet-rich plasma following the addition of ADP as a platelet activator [33]. Since then, there have been major advances in the technologies and methods used. These assays may be used to assess platelets for one or more of their functions, including adhesion, secretion and aggregation. In terms of clinical applications, currently, PFA assays are mainly used in a number of situations such as the assessment of blood coagulability in hospitalized patients before surgery, diagnosis of congenital or acquired platelet dysfunction and monitoring antiplatelet treatment [34].

A sensitive and precise PFA for monitoring antiplatelet treatments would allow clinicians to adjust the drug type or dose (e.g., increase dose, decrease dose, use dual antiplatelet agents or switch to a different drug) to improve the therapeutic outcomes (in this case, decreasing the rate of stroke recurrence). Some researchers are cautiously optimistic about the potential usefulness of standardized PFAs in the development of tailored antiplatelet treatments in patients with cerebrovascular or cardiovascular disease [35,36], while others believe that PFA-guided treatment in stroke patients is currently impractical because of the lack of consensus on the definition of HTPR [13,37], or the lack of a good correlation between PFA results and clinical outcomes [38]. Given these divergent views, the aim of this systematic review was to examine the published evidence for the effectiveness of PFA-based antiplatelet therapy modification (ATM) in patients with ischemic stroke or TIA for the prevention of a recurrent stroke. To the best of our knowledge, this is the first systematic review and meta-analysis undertaken on this topic.

## 2. Materials and Methods

### 2.1. Inclusion and Exclusion Criteria

The study’s inclusion and exclusion criteria are listed in Box 1.

Box 1 **The study’s inclusion and exclusion criteria.**

**Inclusion criteria**:

(1) Full text peer-reviewed journal articles
(2) Clinical trials and observational studies
(3) Published in English, Chinese or Persian (Farsi) languages
(4) Published from inception to 7 January 2020
(5) Adults with ischemic stroke or transient ischemic attack
(6) Patients receiving aspirin and/or clopidogrel were followed up for clinical outcomes for at least 3 months
(7) Platelet function analysis (PFA) results were used for making decisions on the choice of antiplatelet drugs or doses

**Exclusion criteria**:

(1) Not a clinical study (e.g., reviews)
(2) Patients under 18 years of age
(3) Patients with primary diagnosis of coronary or peripheral artery disease
(4) Aspirin or clopidogrel were not administered
(5) Patients were receiving anticoagulants
(6) No PFA-guided antiplatelet drug selection or dose adjustment
(7) Clinical outcomes were not studied
(8) Full text unavailable
(9) Not published in English, Chinese or Persian (Farsi) languages

### 2.2. Participants

Patients with a preliminary diagnosis of ischemic stroke or minor stroke (TIA) who underwent aspirin or clopidogrel therapy following the initial diagnosis were included.

### 2.3. Types of Interventions

Types of interventions included in the review were PFA-guided modifications in antiplatelet therapies (including increasing the drug dose, adding another antiplatelet drug and switching to another antiplatelet agent), compared to standard antiplatelet therapies based on the current clinical guidelines [3,5], which do not recommend the use of PFA for therapeutic decision making.

### 2.4. Types of Outcome Measures

Primary outcomes were recurrence of stroke or TIA, and secondary outcomes were death and/or bleeding incidences.

### 2.5. Search Methods

The systematic review was registered on the International Prospective Register of Systematic Reviews (PROSPERO) (registration ID: CRD42019126946; https://www.crd.york.ac.uk/prospero/display_record.php?ID=CRD42019126946). Full-text peer-reviewed journal articles were searched through five online databases (Embase (Scopus), Cochrane Library, Medline, CINAHL and Web of Science) for articles published in English, Chinese or Persian languages from inception of the databases to 7 January 2020. Different combinations of the following search terms were used: aspirin, clopidogrel, antiplatelet, stroke, cerebrovascular disease, transient ischemic attack, TIA, large-artery atherosclerosis, LAA, platelet function analysis, platelet aggregation, PFA-100, PFA-200, VerifyNow, Multiplate, aggregometry, aspirin resistance, platelet reactivity, clopidogrel resistance, high on-treatment platelet reactivity, HTPR, platelet residual activity, platelet hyperactivity, aspirin non-responder, and clopidogrel non-responder.

### 2.6. Quality Assessment and Publication Bias

The included observational studies were assessed using the Newcastle-Ottawa Scale (NOS) [39]. For cohort studies, NOS includes the following domains: (1) selection of the exposed cohort and the non-exposed cohort with ascertainment of exposure and demonstration that the outcome of interest was not present at the start of the study, (2) comparability of cohorts on the basis of the design or analysis and (3) assessment of outcome, and adequate follow-up time and rate [39].

### 2.7. Data Extraction

The following data were extracted: authors; year of publication; sample size; patient diagnosis and demographics; antiplatelet regimen including medication, dosage, duration and any alterations; platelet function test values and cut-off values; therapeutic window of platelet reactivity for antiplatelet regimen adjustment; and prevalence or relative risk or odds risk of secondary stroke.

### 2.8. Data Analysis

Review Manager 5 software (Copenhagen: The Nordic Cochrane Centre.; version 5.4, The Cochrane Collaboration, London, UK) was employed in all analytic processes. Odds ratios (OR) with 95% confidence intervals (95% CI) of recurrent ischemic stroke were generated to determine the pooled effect of modification in antiplatelet therapy. Heterogeneity was explored by using the chi-square test, with a *p*-value of < 0.10 indicating significant heterogeneity. Inconsistency across studies was then quantified with the *I*^2^ statistic test, with an *I*^2^ value between 50% and 75% indicating moderate heterogeneity, and a value of >75% indicating high heterogeneity. Fixed effects were carried out with low levels of clinical or statistical heterogeneity, and random effects were used when the heterogeneity was above 50%.

We analyzed the overall effects of modification in antiplatelet therapy compared to aspirin and/or clopidogrel treatments without adjustment, and the effects of modification in antiplatelet therapy in aspirin non-responders [40,41]. The data for clopidogrel non-responders were not included in the meta-analysis, because they were reported only in one study [40].

## 3. Results

### 3.1. Study Selection

**Figure 1:**
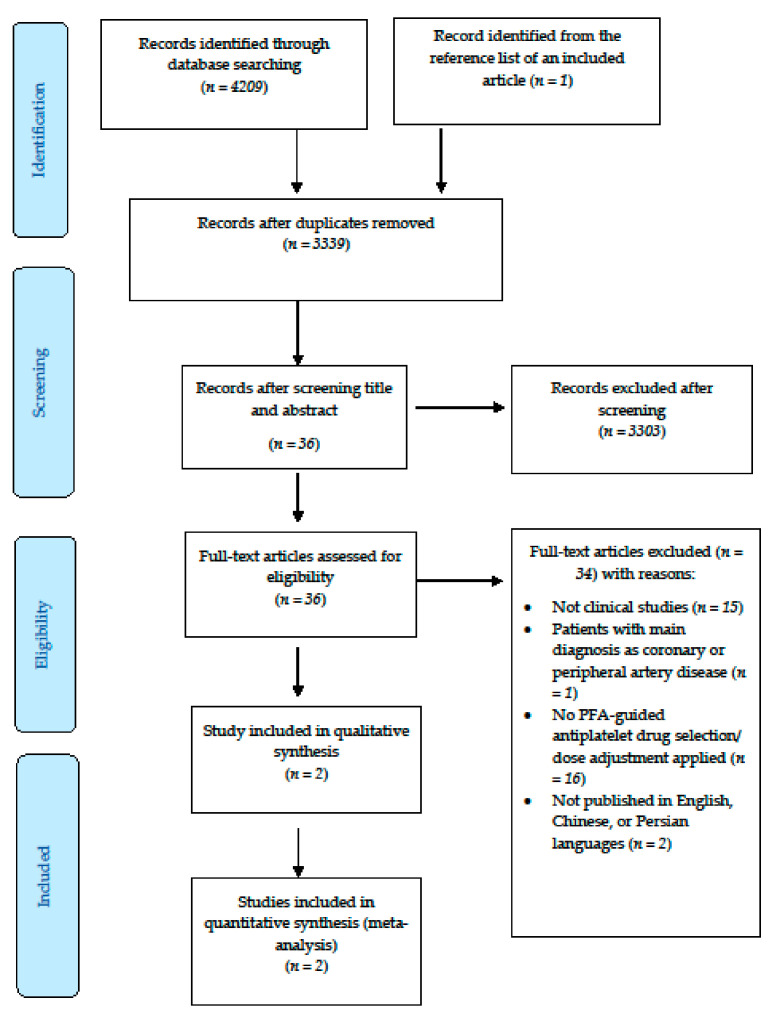
The processes of the study based on the Preferred Reporting Items for Systematic Reviews and Meta-Analyses (PRISMA) 2009 Flow Diagram.

Figure 1 depicts the search process for this study using the Preferred Reporting Items for Systematic Reviews and Meta-Analyses (PRISMA) 2009 Flow Diagram. We were able to find only two observational studies which met our inclusion criteria [40,41].

### 3.2. Characteristics of the Studies

One of the included studies were undertaken in a medical center in the US [40] and the other study in three centers in China [41]. Altogether, these two studies examined 1136 participants who were on antiplatelet therapy after a diagnosis of ischemic stroke or TIA. Depta et al. [40] conducted the comparison in mixed aspirin and/or clopidogrel users, while the study by Yi et al. [41] included patients with aspirin monotherapy before platelet function testing. The accumulated rate of recurrent stroke and treatment side effects was observed within a mean follow-up period of 4.6 ± 1.1 years and 4.8 ± 1.7 years, respectively. The study designs and the characteristics of the participants, interventions and outcome measures are presented in Table 1 and Table 2.

### 3.3. Comparisons

In both studies, the participants were originally prescribed an antiplatelet for the prevention of recurrent thrombotic events, and antiplatelet therapy modification was defined as any changes in antiplatelet regimen within 24 h after platelet function testing. However, the two studies varied in original antiplatelet therapy and the specific modification in antiplatelet regimens. Yi et al. [41] studied aspirin monotherapy and four types of modification: (1) changed from aspirin to clopidogrel, (2) changed from aspirin to cilostazol, (3) increased aspirin doses and (4) added clopidogrel to aspirin. Depta et al. [40] studied aspirin and/or clopidogrel treatment and seven types of modification: (1) added or increased aspirin doses, (2) added aspirin, (3) added aspirin/dipyridamole, (4) added or increased clopidogrel, (5) added clopidogrel, (6) increased or added both aspirin or clopidogrel, and (7) changed from aspirin to clopidogrel. The comparison of the rates of recurrent stroke was conducted in overall patients and subgroups (i.e., aspirin non-responders and/or clopidogrel non-responders) between those with antiplatelet modification (ATM) and without ATM.

### 3.4. Outcomes

Both studies recorded ischemic events (ischemic stroke, transient ischemic attack and myocardial infarction), any bleeding events and deaths from any cause.

### 3.5. Quality

The studies had similar methodologies, but there were some improvements in the study by Yi et al. [41]. Although the non-exposure cohort (without ATM) were drawn from the same register as the exposed cohort (with ATM), potential selection bias, caused by unknown clinical factors that may affect physicians’ decisions regarding platelet function test results and antiplatelet regimens, existed in both studies. For exclusion of cases in which the study outcome (i.e., recurrent stroke) had already occurred at the start of the study, Yi et al. [41] included only the first-ever ischemic stroke patients, while Depta et al. [40] did not.

In terms of comparability, both studies conducted adjustments for propensity scores, which included age, male, inpatient and risk factors for stroke, such as smoking status, diabetes, hypertension, prior cardiovascular disease and surgical treatment, as well as history of medications like antiplatelet, antihypertensive and hypoglycemic agents. However, adherence to the antiplatelet therapy was not assessed in either of the studies. The diagnosis of ischemic stroke subtypes was undertaken only in the study by Yi et al. [41]. Neither of the studies described the subjects lost to follow-up in any detail.

### 3.6. The Overall Effects of Modified Antiplatelet Therapy

**Figure 2:**
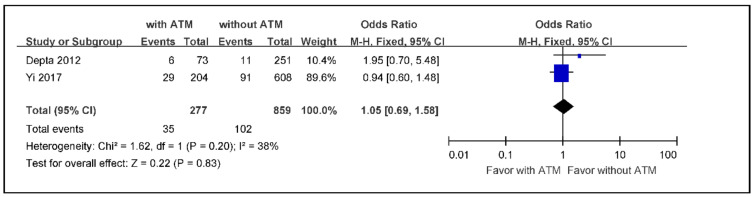
The meta-analysis of the incidence rate of recurrent ischemic stroke in ischemic stroke or TIA patients with ATM versus those without ATM (*n* = 1136). TIA: transient ischemic attack, ATM: antiplatelet therapy modification.

The meta-analysis of the incidence rates of recurrent ischemic stroke in ischemic stroke or TIA patients with ATM versus those without ATM, using a fixed effects model because of low heterogeneity, indicated an overall effect size of 0.22 without statistical significance (OR 1.05; 95% CI 0.69 to 1.58) (Figure 2).

**Figure 3:**
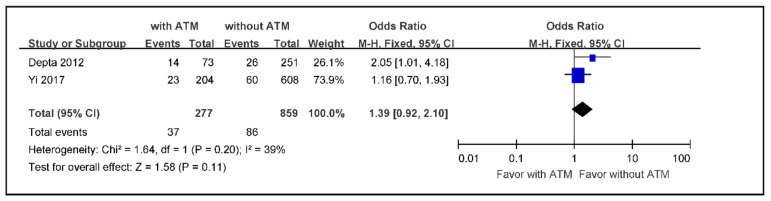
The meta-analysis of the incidence rate of bleeding in ischemic stroke or TIA patients with ATM versus those without ATM (*n* = 1136). TIA: transient ischemic attack, ATM: antiplatelet therapy modification.

The meta-analysis of the incidence rate of any bleeding in ischemic stroke or TIA patients with ATM versus those without ATM, using a fixed effects model because of low heterogeneity, indicated an overall effect size of 1.58 without statistical significance (OR 1.39; 95% CI 0.92 to 2.10) (Figure 3).

**Figure 4:**
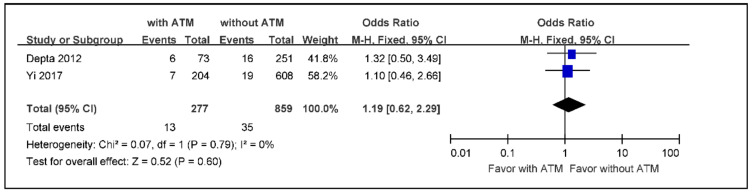
The meta-analysis of the incidence rate of death in ischemic stroke or TIA patients with ATM versus those without ATM (*n* = 1136). TIA: transient ischemic attack, ATM: antiplatelet therapy modification.

The meta-analysis of the incidence of death from any cause in ischemic stroke or TIA patients with ATM versus those without ATM, using a fixed effects model because of low heterogeneity, indicated an overall effect size of 0.52 without statistical significance (OR 1.19; 95% CI 0.62 to 2.29) (Figure 4).

### 3.7. Effect of Modified Antiplatelet Therapy in Aspirin Non-Responders

**Figure 5:**
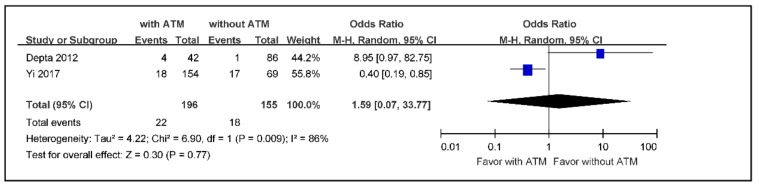
The meta-analysis of the incidence rate of recurrent ischemic stroke in ischemic stroke or TIA aspirin non-responders with ATM versus those without ATM (*n* = 351). TIA: transient ischemic attack, ATM: antiplatelet therapy modification.

The subgroup meta-analysis of the incidence rate of recurrent ischemic stroke in ischemic stroke or TIA aspirin non-responders with ATM versus those without ATM, using a random effects model because of high heterogeneity, indicated an effect size of 0.30 without statistical significance (OR 1.59; 95% CI 0.07 to 33.77) (Figure 5).

**Figure 6:**
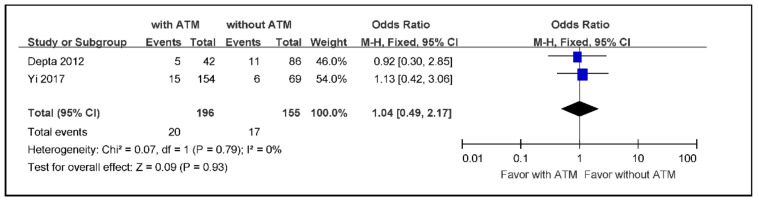
The meta-analysis of the incidence rate of bleeding in ischemic stroke or TIA aspirin non-responders with ATM versus those without ATM (*n* = 351). TIA: transient ischemic attack, ATM: antiplatelet therapy modification.

The subgroup meta-analysis of the incidence rate of any bleeding in ischemic stroke or TIA aspirin non-responders with ATM versus those without ATM, using a fixed effects model because of low heterogeneity, indicated an effect size of 0.09 without statistical significance (OR 1.04; 95% CI 0.49 to 2.17) (Figure 6).

**Figure 7:**
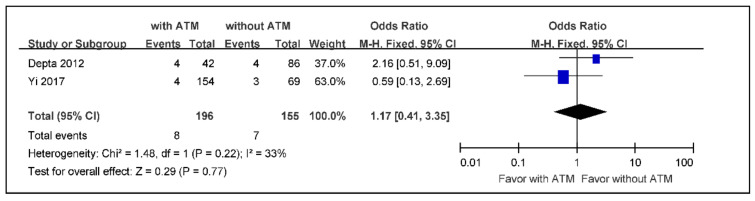
The meta-analysis of the incidence rate of death in ischemic stroke or TIA aspirin non-responders with ATM versus those without ATM (*n* = 351). TIA: transient ischemic attack, ATM: antiplatelet therapy modification.

The subgroup meta-analysis of the incidence rate of death from any cause in ischemic stroke or TIA aspirin non-responders with ATM versus those without ATM, using a fixed effects model because of low heterogeneity, indicated an effect size of 0.29 without statistical significance (OR 1.17; 95% CI 0.41 to 3.35) (Figure 7).

## 4. Discussion

The analyses of the pooled data indicated that, compared with standard antiplatelet therapy (i.e., without ATM), the overall effects of PFA-guided ATM on recurrent strokes, any bleeding risk or death hazard were not statistically significant, although the group with ATM had a significantly higher residual platelet reactivity than the group without ATM. Higher residual platelet reactivity has been known as an independent risk factor for recurrent stroke in patients with ischemic stroke or transient ischemic attack [19], but ATM was successful in keeping the rate of recurrent ischemic stroke for ischemic stroke or TIA patients with higher residual platelet reactivity down to the same value as for the antiplatelet responders.

Modification in antiplatelet therapy was associated with an increased risk for any bleeding event in the study by Depta et al. [40] (19% vs. 10%, *p* = 0.04), while there was no significant change in the rate for any bleeding event after antiplatelet therapy modification in the study by Yi et al. [41] (11.3% vs. 9.9%, *p* = 0.61). Moreover, the effects of PFA-guided ATM on the risk of recurrent ischemic stroke in the subgroup of aspirin non-responders were opposite, leading to a result without statistical significance in the meta-analysis. In one study (Yi et al. [41]), it was reported that the antiplatelet therapy modification significantly lowered the recurrence rate of ischemic stroke (11.7% vs. 24.6%, *p* = 0.02), whereas the other study (Depta et al. [40]) reported an increase in the recurrence rate of ischemic stroke by antiplatelet therapy modification with borderline significance (10% vs. 1%, *p* = 0.04).

To be able to justify these kinds of inconsistencies between the two studies, they should be looked at from different perspectives. Firstly, the predictive value of HTPR for clinical outcomes may be complicated because of multiple etiologies [42], as the roles of the platelet reactivity may be different in different vascular diseases (cardiovascular versus cerebrovascular) [43], or even different subtypes of ischemic stroke [44,45,46]. Between the two included studies in this systematic review, this is only the study by Yi et al. [41] which identifies the stroke subtypes in the patients. The study sample was more homogeneous in the study by Yi et al. [41], as only two subtypes were included (i.e., the atherothrombotic and small artery disease). Although small artery disease could be thrombotic or embolic, cerebral embolism was excluded in this study.

Additionally, the prevalence of aspirin non-response in the study by Depta et al. [40] was much higher than in the study by Yi et al. [41] (43% vs. 27.5%), while both studies adopted the same technology (optical platelet aggregometry) for platelet function analysis. The latter included patients with first-time stroke only, while the former did not clarify this. Hence, the study by Depta et al. [40] may have enrolled patients with recurrent stroke, and it is known that patients with prior stroke or TIA have an increased risk for recurrent stroke [47].

Regardless of the above inconsistencies, both studies had limitations in controlling the potential confounders, which should be taken into consideration in future studies. Firstly, neither of the studies did report the patient adherence to antiplatelet treatment, which could be a confounder in assessing the efficacy of antiplatelet agents, and in evaluating the effect of true HTPR compared to pseudo HTPR (due to non-compliance). This is probably a common issue in antiplatelet treatments, as Dawson et al. [48] reported a 60% patient non-adherence rate following the urinary measurement of aspirin metabolites. In addition, the reported drop of nearly 50% in the HTPR rates in two studies of stroke patients following the supervised administration of aspirin indicates the role of patients’ non-compliance in influencing HTPR results [49,50].

Secondly, the proportion of patients undergoing platelet function re-testing after antiplatelet therapy modification was quite low in both studies. Not only the platelet function re-testing can be used for assessing the effectiveness of the modified antiplatelet therapies [51], but also it can help detect a sustained HTPR as a risk factor for recurrent stroke. Accounting for the dynamic feature of HTPR may be essential for optimizing the protocols for platelet function analyses and establishing specific criteria for the frequency of retesting and the choice of antiplatelet therapy modification [13,52]. Although the included studies involved the same method of laboratory testing, it is necessary to understand that the laboratory identification of HTPR depends on assay-specific factors such as the exact method, the device, and the cut-off values used [13]. As a result, more research should be done to rectify these technical issues so that PFA can be used consistently in different clinical practices.

Thirdly, although HTPR can be, in some cases, improved by either increasing the antiplatelet dose [20,43,53] or adding another type of platelet inhibitor [54], the pharmacological response to an antiplatelet therapy (i.e., clinical responsiveness) may not be exactly the same phenomenon that is measured through laboratory testing. In other word, the concepts of clinical resistance and laboratory-measured resistance may be quite different [55].

As our study limitation, we could not find any randomized controlled clinical trials or prospective cohort studies to meet our inclusion criteria, so we had to include only two retrospective cohort studies with relatively small sample sizes. This affects the power of our meta-analysis and the generalizability of the results. However, as mentioned in the discussions above, due to the scarcity of clinical studies in this area, and given the serious consequences of recurrent stroke, there is a strong need for more research in this area to find ways to improve the effectiveness of antiplatelet treatments in stroke patients.

## 5. Conclusions

Given the small number of participants in the included studies and the lack of randomized clinical trials in this area, it is not certain whether a PFA-guided antiplatelet therapy would be successful in improving patient outcomes by decreasing the rates of secondary stroke while minimizing the risk of bleeding. Thus, well-designed randomized controlled trials are needed to obtain stronger evidence to address the research question.

## Figures and Tables

**Table 1:** The study designs and methods of the included studies.

Study	Patient and Sample Size			Intervention	Comparison	Main Outcomes	Follow-Up Time (Mean ± SD)
	Overall	Subgroup 1	Subgroup 2				
Depta et al. [40]	ischemic stroke or TIA (*n* = 324)	aspirin non-responders * (*n* = 128)	clopidogrel non-responders ^#^ (*n* = 54)	ATM ^a^	aspirin and/or clopidogrel treatment	recurrence of ischemic stroke bleeding death	4.6 ± 1.1 years
Yi et al. [41]	first-ever ischemic stroke with two subtypes of stroke: atherothrombotic or small artery disease (*n* = 812)	aspirin non-responders * (*n* = 223)	not studied	ATM ^b^	aspirin monotherapy	recurrence of ischemic stroke bleeding death	4.8 ± 1.7 years

SD: standard deviation, ATM: antiplatelet modification, TIA: transient ischemic attack. * ≥20% aggregation with 0.5% mg/mL arachidonic acid (AA), or ≥70% aggregation with 10 µM adenosine diphosphate (ADP), or on-aspirin onset of ischemic stroke or TIA. ^#^ ≥70% aggregation with 10 µM ADP. ^a^ Seven types of modification: added or increased aspirin, added aspirin, added aspirin/clopidogrel, added or increased clopidogrel, added clopidogrel, increased or added both aspirin and clopidogrel, changed from aspirin to clopidogrel. ^b^ Four kinds of modification: changed from aspirin to clopidogrel, changed from aspirin to cilostazol, increased aspirin, added clopidogrel to aspirin.

**Table 2:** Characteristics and outcomes of the included studies.

Study	Intervention and Patient Characteristics Mean ± SD	Main Outcomes					
		Recurrent Ischemic Stroke	*p*-Value	Bleeding	*p*-Value	Death	*p*-Value
Depta et al. [40]	With ATM ^a^ age: 71.4 ± 11.9 years aggregation with AA, %: 26.7 ± 19.7 aggregation with ADP, %: 56.2 ± 22.9	6/73 (8%)	0.23	14/73 (19%)	0.04	6/73 (8%)	0.60
	Without ATM ^a^ age: 65.6 ± 13.5 years aggregation with AA, %: 19.1 ± 14.0 aggregation with ADP, %: 46.5 ± 23.5	11/251 (4%)		26/251 (10)		16/251 (6%)	
Yi et al. [41]	With ATM ^b^ age: 71.8 ± 11.6 years aggregation with AA, %: 26.8 ± 10.2 aggregation with ADP, %: 58.4 ± 18.6	29/204 (14.2%)	0.82	23/204 (11.3%)	0.61	7/204 (3.4%)	0.84
	Without ATM ^b^ age: 67.1 ± 13.6 years * aggregation with AA, %: 20.1 ± 8.7 * aggregation with ADP, %: 47.6 ± 16.4	91/608 (15.0%)		60/608 (9.9%)		19/608 (3.1%)	
**Study**	Subgroup: Aspirin Non-Responders *^,a^	**Main Outcomes**					
		**Recurrent Ischemic Stroke**	***p*-Value**	**Bleeding**	***p*-Value**	**Death**	***p*-Value**
Depta et al. [40]	With ATM ^a^ Patient characteristics not stated	4/42 (10%)	0.04	5/42 (12%)	0.89	4/42 (10%)	0.44
	Without ATM ^a^ Patient characteristics not stated	1/86 (1%)		11/86 (13%)		4/86 (5%)	
Yi et al. [41]	With ATM ^b^ Patient characteristics not different significantly	18/154 (11.7%)	0.008	15/154 (9.7%)	0.81	4/154 (2.6%)	0.67
	Without ATM ^b^ Patient characteristics not different significantly	17/69 (24.6%)		6/69 (8.7%)		3/69 (4.3%)	

SD: standard deviation, ATM: antiplatelet modification, AA: arachidonic acid, ADP: adenosine diphosphate. * Aspirin non-responsiveness was defined as ≥20% aggregation with 0.5% mg/mL AA, or ≥70% aggregation with 10 µM ADP, or on-aspirin onset of ischemic stroke or TIA. ^a^ Seven types of modification: added or increased aspirin, added aspirin, added aspirin/clopidogrel, added or increased clopidogrel, added clopidogrel, increased or added both aspirin and clopidogrel, changed from aspirin to clopidogrel. ^b^ Four kinds of modification: changed from aspirin to clopidogrel, changed from aspirin to cilostazol, increased aspirin, added clopidogrel to aspirin.
